# Neurturin Evokes MAPK-Dependent Upregulation of Egr4 and KCC2 in Developing Neurons

**DOI:** 10.1155/2011/641248

**Published:** 2011-07-04

**Authors:** Anastasia Ludwig, Pavel Uvarov, Christophe Pellegrino, Judith Thomas-Crusells, Sebastian Schuchmann, Mart Saarma, Matti S. Airaksinen, Claudio Rivera

**Affiliations:** ^1^Neuroscience Center, University of Helsinki, Viikinkaari 4, 00014 Helsinki, Finland; ^2^Institute of Biotechnology, University of Helsinki, Viikki Campus, Viikinkaari 9, 00014 Helsinki, Finland; ^3^Inserm Unité 901, 13009 Marseille, France; ^4^UMR S901 Aix-Marseille 2, Université de la Méditerranée, 13009 Marseille, France; ^5^INMED/INSERM u901, 13009 Marseille, France; ^6^Neuroscience Research Center, Charité-Universitätsmedizin, 10117 Berlin, Germany; ^7^Experimental Animal Center, University of Helsinki, 00014 Helsinki, Finland

## Abstract

The K-Cl cotransporter KCC2 plays a crucial role in the functional development of GABA_A_-mediated responses rendering GABA hyperpolarizing in adult neurons. We have previously shown that BDNF upregulates KCC2 in immature neurons through the transcription factor Egr4. The effect of BDNF on Egr4 and KCC2 was shown to be dependent on the activation of ERK1/2. Here we demonstrate that the trophic factor neurturin can also trigger Egr4 expression and upregulate KCC2 in an ERK1/2-dependent manner. These results show that Egr4 is an important component in the mechanism for trophic factor-mediated upregulation of KCC2 in immature neurons involving the activation of specific intracellular pathways common to BDNF and Neurturin.

## 1. Introduction

The maturation of GABA_A_ mediated neurotransmission encompasses long-term qualitative changes in postsynaptic responses. Particularly important is the developmental shift from depolarizing to hyperpolarizing GABA_A_-evoked responses. This transition is attributed to the increase in expression of the K-Cl cotransporter KCC2. The functional expression of this cotransporter keeps the intraneuronal chloride concentration below that predicted from passive distribution rendering the GABA_A_ reversal potential more negative than the resting membrane potential [1].

We have shown previously that BDNF-mediated TrkB activation can regulate the expression of KCC2 differentially in adult and in early postnatal neurons [2–4]. Activation of TrkB by BDNF triggers two major intracellular cascades [5–8], namely, the Shc and the PLC*γ* pathways. (i) The Shc pathway includes the activation of a number of adaptor proteins such as Shc, SOS, and Grb2 that results in the GTP-loading of Ras that subsequently leads to the activation of the MAP kinase cascade. This comprises the sequential phosphorylation of Raf, Mek, and Erk. The phosphorylated Erk is then translocated to the nucleus where it can activate and induce the expression of transcription factors. (ii) In the PLC*γ* pathway, direct activation of PLC*γ* by TrkB leads to the breakdown of PIP2 into DAG and IP_3_. Activation of PKC by DAG promotes the release of intracellular Ca^2+^ and the activation of Ca^2+^-dependent proteins. Interestingly, although BDNF induces under normal physiological conditions downregulation of KCC2 in adult neurons, it can induce KCC2 up-regulation if TrkB activation specifically evokes signaling through the Shc pathway only [2, 4]. Also in immature neurons BDNF can induce KCC2 up-regulation in an MAPK-dependent manner acting though the transcription factor Egr4 [3] suggesting that BDNF/TrkB-mediated upregulation of KCC2 use intracellular pathways downstream of Shc.

Other neurotrophic factors, can also trigger Shc/MAPK signaling. Neurturin belongs to the GDNF family of neurotrophic factors and it specifically binds to the GPI-anchored receptor GRF*α*2. The ligand binding triggers association of GFR*α*2 with transmembrane tyrosine-kinase receptor RET that in turn can activate signaling pathways including the Shc/MAPK intercellular cascades [9]. 

In the present report, we study the effect of Neurturin on the regulation of KCC2 at the transcriptional level. We found that Neurturin can trigger the expression of Egr4. Accordingly, Neurturin induces the activation of the KCC2 proximal promoter region that results in a significant increase in KCC2 protein expression in immature neurons. Most importantly, this requires MEK-dependent ERK phosphorylation. We also show that Neurturin can evoke up-regulation of KCC2 *in vivo* after a single intrahippocampal application. Taking into account that BDNF, acting through similar intracellular signaling cascades, is able to up-regulate KCC2 in immature neurons, we propose a general mechanism for trophic factor-mediated KCC2 gene regulation in immature neurons, where Egr4 downstream of the MAPK/ERK signaling pathway plays a crucial role. 

## 2. Methods

### 2.1. Dissociated Cultures

All animal experiments were approved by the local ethics committee for animal research at the University of Helsinki. Standard dissociated hippocampal cultures were prepared from embryonic day 17 (E17) mice as described in the original protocol [10], with slight modifications. Briefly, a pregnant mouse was anaesthetized in a CO_2_ chamber and sacrificed by cervical dislocation, embryos were removed, and hippocampi were dissected. Cells were dissociated by enzymatic treatment (0.25% trypsin for 15 min at 37°C) and plated on poly-DL-ornithine-coated coverslips (50000 cells/cm^2^) in neurobasal medium containing B27 supplement (Gibco, Life Technologies). Before plating, the medium was preincubated on astroglial culture for 24 hours. Neuronal cultures were fed once a week by changing half of the medium. Astroglial cultures were prepared according to Banker and Goslin, 1998, and maintained in DMEM supplemented with 10% of foetal calf serum, penicillin 100 units/mL, and streptomycin 100 *μ*g/mL.

### 2.2. Organotypic Cultures

Hippocampal organotypic cultures were prepared according to the method of Stoppini [11]. Transverse slices (thickness 350 *μ*m) were cut from the hippocampi from P8 mice using a McIlwain tissue chopper. They were immediately placed on sterile Millicell-CM membranes (Millipore) in 6-well culture trays with 1 mL of plating medium. The plating medium was neurobasal medium containing B27 supplement (Gibco, Life Technologies), penicillin 100 units/mL and streptomycin 100 *μ*g/mL. One day after plating the medium was changed to the growth medium Neurobasal/B27 without antibiotics. The cultures were grown at 37°C under 5% CO_2_ in air, and the medium was changed twice a week.

### 2.3. Application of Growth Factors

Treatments of organotypic cultures were performed from div (day *in vitro*) 2 until div5. Dissociated cultures were treated with Neurturin during div1–div4, div8–div11, and div15–18. Neurturin (PeproTech Inc.) was added from the frozen stock once on the first day of the treatment period. The final concentration of Neurturin is indicated in Section 3.

### 2.4. Western Blotting

Neuronal cultures were rinsed in PBS, scraped into ice-cold lysis buffer (NaCl 150 mM; TritonX-100 1%; Doc 0.5%; SDS 0.1%; TrisHCl 50 mM  pH 8.0) and homogenized. Hippocampal slices were homogenized directly with lysis buffer. Protein concentrations were determined using D_C_ Protein Assay kit (Bio-Rad). Samples were separated using 7.5% SDS-PAGE and transferred onto Hybond ECL nitrocellulose membrane (Amersham, Pharmacia Biotech). Blots were probed with anti-KCC2 rabbit polyclonal antibody [12] at 1 : 5000 dilution and anti-*β*-tubulin rabbit polyclonal antibody (Covance, PRB-435P, 1 : 3000), developed with ECL-plus (Amersham, Pharmacia Biotech), and visualized with luminescent image analyzer LAS-3000 (Fujifilm). Optical densities of the bands were analyzed with AIDA imaging software (Raytest).

### 2.5. Semi-Quantitative RT-PCR

Neuronal dissociated cultures treated with growth factors were used for total RNA isolation and reverse transcription reaction. Total RNA was isolated using RNeasy Mini Kit (Qiagen) according to manufacturer's instructions. Isolated RNA samples were reverse transcribed using random hexamer primers and Superscript II reverse transcriptase (Life Technologies). The samples after reverse transcription were diluted 1/10, 1/20, 1/50, 1/100, and amplified for 30–34 cycles using DyNAzyme EXT polymerase (Finnzymes) to keep the product amplification in the exponential range for every primer pair (see Table 1).

The PCR conditions for all primer pairs were the same: 2 min of the initial denaturation at 95°C followed by 30–34 cycles with 95°C for 30 sec, 55°C for 30 sec, and 72°C for 1 min. Products were analyzed on 1.5% agarose gel and visualized with luminescent image analyzer LAS-3000 (Fujifilm). Optical densities of the bands were analyzed with AIDA imaging software (Raytest).

### 2.6. Quantitative RT-PCR Analysis

Total RNA was isolated with the RNeasy Micro (Qiagen) kit. Typically, about 1 *μ*g of total RNA was reverse transcribed using the SuperScript III Reverse Transcriptase (Life Technologies) and random primers (at 37°C) according to the manufacturer's protocol. The cDNA samples were amplified using the SYBR Green PCR Master Mix (Applied Biosystems) and detected via the ABI Prism 7000 Sequence Detection System (Applied Biosystems). Primers for Egr4 and glyceraldehyde-3-phosphate dehydrogenase (GAPDH) quantification were designed with the Express v2.0 software (Applied Biosystems) and contained, when possible, intronic sequence in between (see Table 2).

### 2.7. Transfection and Luciferase Assay

The neurons were transfected with the luciferase reporter construct using Lipofectamine 2000 (Life Technologies) according to the manufacturer's protocol at div5. To avoid cytotoxicity, we used relatively low amounts of the luciferase construct (0.5 *μ*g per 1-cm-diameter well). Two days after transfection the neurons were briefly washed with phosphate-buffered saline (PBS) and lysed in Passive Lysis Buffer (Promega). Renilla and Firefly luciferase activities were measured with a Dual-Luciferase Reporter Assay System according to the manufacturer's protocol.

### 2.8. In Vivo Injections of Neurturin

Neurturin (1 *μ*g) was injected in hippocampus of P5-P6 rats. Animals were hypothermically anesthetized and heads were fixed in a surgical mask to maintain the skull stable. A midline incision was made on the head, and the hole was drilled in the skull. The stereotactic coordinates for injection were anteroposterior-1.8 mm (relative to the bregma), mediolateral 2 mm (relative to the bregma), dorsoventral-2 mm from the cortical surface. Neurturin was dissolved in 4 *μ*L of saline solution (123 mM NaCl, 5 mM KCl, 1,25 mM NaH_2_PO_4_, 2 mM MgSO_4_, 10 mM glucose, 2 mM CaCl_2_, 10 mM HEPES, and pH 7.2) and injected in right-side hippocampus at ~1 *μ*L/min. Similarly left-side hippocampus was injected with 4 *μ*L of pure saline solution. After injection, the needle was left in the tissue for 2 minutes. The incision was sutured and the rat was allowed to fully recover before being placed back with littermates.

### 2.9. Image Analysis

For each injected brain a series of coronal hippocampal sections (see immunohistochemistry) was made at P8-P9, three days after injection. The sections in the series were numbered in succession starting from most frontal part of hippocampus. Each section contained Neurturin-injected side (right) as well as control side (left). KCC2 levels were analyzed by immunostaining in sections number 45, 90, 132, 177, 222, and 273. The section thickness was 7 *μ*M which gives a sample interval of approximately 315 *μ*m. In each brain analyzed, the injection site (defined by a scar) was found between section number 60 and section number 90 and located in right and left hemisphere in CA1 area of hippocampus. 

Confocal images of immunostained tissue were made. For each image nine consecutive optical slices (0.8 *μ*m) were made and merged for quantification of KCC2 signal intensity. The region of interest was manually highlighted and total intensity of immunostaining in the region was divided by its area. KCC2 intensity in Neurturin-injected hemisphere was normalized to the KCC2 intensity in corresponding area of the same section control hemisphere.

### 2.10. Immunohistochemistry

Rats were deeply anaesthetized with pentobarbital and perfused with 4% PFA in PBS. Brains were removed and stored overnight in 4% PFA in PBS at 4°C. Tissues were paraffin embedded and cut into 7-*μ*m-thick sagittal (*in situ*) or coronal (*in vivo* injections) sections. Deparaffinized sections were washed with 1% SDS in TBST (0.1% Tween in TBS), treated with 100 *μ*g/mL saponin in TBST for 30 min at room temperature, and then treated with 5% bovine serum albumin (BSA) in TBST for two hours at room temperature. Next, tissues were incubated with anti-KCC2 rabbit polyclonal antibody [12] diluted at 1:5000 in 2% BSA, 0.2% Triton X-100, TBST overnight at 4°C. Species-specific secondary antibodies: donkey antirabbit Cy3 (Jackson Laboratories, 711-166-152), donkey anti-rabbit Alexa Fluor 488, and goat antiguinea pig Alexa Fluor 488 (Molecular Probes, Invitrogen, catalog number: A-21206, A-11073, resp.) were used at 1 : 400 dilution. Sections were visualized with Leica TCS SP2 AOBS confocal system.

### 2.11. In Situ Hybridization

*In situ* hybridization on paraffin-embedded sections was done as described [13]. Sagittal sections (thickness 7 *μ*m) were hybridized using ^35^S-labelled antisense and sense (control) cRNA probes: RET-specific probe (nucleotides 2595–3191, X67812) and GFR*α*2 (full length, AF003825) [14]. No labeling above background was observed in the sense controls.

### 2.12. Statistics

The data represents the mean ± SEM. Statistical analysis was performed using one sample *t*-test in GraphPad Prism statistical software. Statistical significance was defined as *P < 0.05, **P < 0.01, ***P < 0.001;  *n* represents the number of independent experiments.

## 3. Results

### 3.1. Neurturin Upregulates KCC2 Expression in Developing Organotypic and Dissociated Hippocampal Cultures

**Figure 1:**
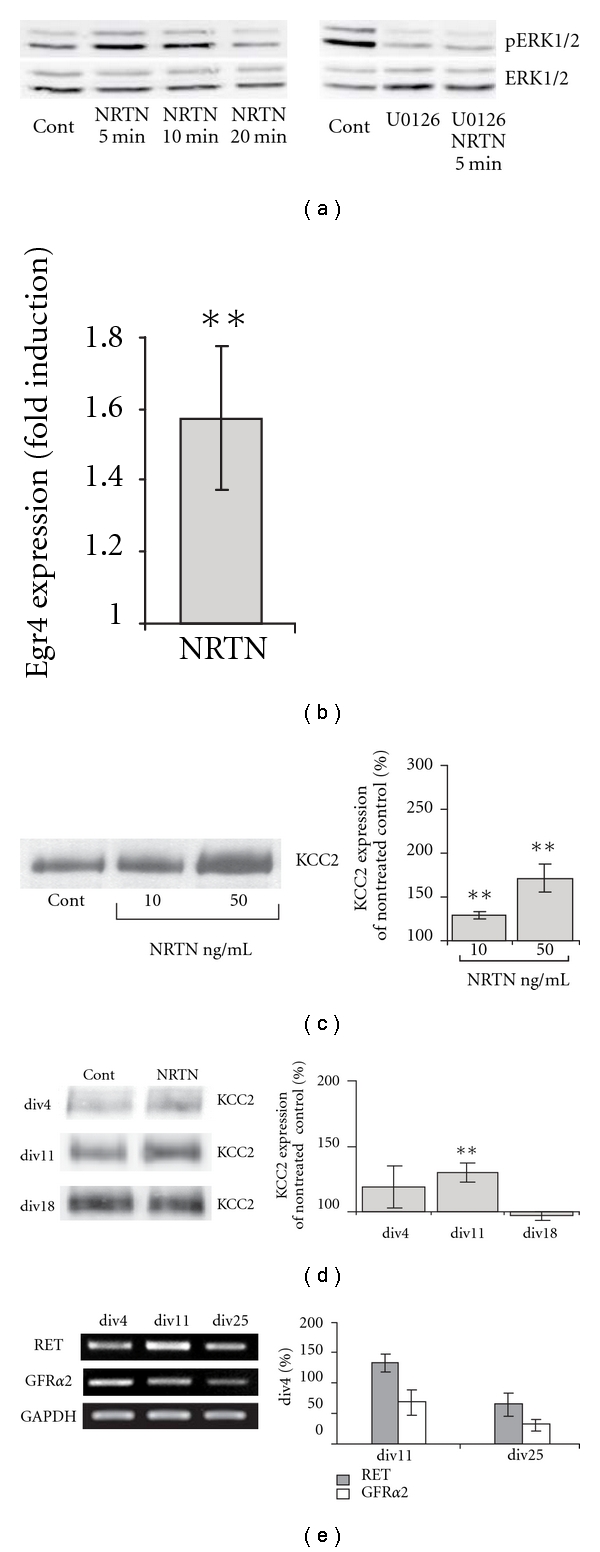
Regulation of KCC2 expression by Neurturin. (a) Representative Western blot analysis of Neurturin-induced ERK1/2 phosphorylation. Lysates of div5-dissociated cultures were collected 5–20 min after Neurturin (50 ng/mL) application. In some cases cultures were pretreated with MEK inhibitor U0126 (20 *μ*M) 30 min before Neurturin application. (b) Egr4 mRNA level in div5–div10 dissociated cultures 1-2 hours after Neurturin (50 ng/mL) application as detected by real time PCR (*n* = 5). Nontreated control value was set to 1. (*P < 0.05, **P < 0.01, ***P < 0.001). Error bars represent SEM. (c) Representative Western blot analysis and quantification of KCC2 expression in organotypic hippocampal slice cultures treated with NTRN (*n *= 4–8). Organotypic cultures were treated with 10 ng/mL and 50 ng/mL Neurturin. Data are normalized to the value in nontreated controls (*P < 0.05, **P < 0.01, ***P < 0.001). Error bars represent SEM. (d) Representative Western blot analysis and quantification of KCC2 expression in dissociated hippocampal cultures treated with Neurturin (50 ng/mL) (*n *= 3–7). Dissociated cultures were treated with Neurturin at div1, div8 and div15 and analyzed 3 days after the treatment. Data are normalized to the value of non-treated controls of the corresponding age (*P < 0.05, **P < 0.01, ***P < 0.001). Error bars represent SEM. (e) Representative semiquantitative RT PCR from cDNA of different age cultures for RET, GFR*α*2, and GAPDH (used as internal standard) and summarized results of 3 similar PCRs. The data show that dissociated hippocampal neurons express detectable levels of growth factors receptors at all ages tested. Data are normalized to div4 value. Error bars represent SEM.

Our previous results provided evidence that Egr4 and KCC2 expression is regulated through ERK1/2-dependent mechanism [3]. This may imply that other means of MAPK activation might lead to the induction of KCC2 expression. Thus we analyzed the effect of Neurturin, another trophic factor that also induces MAPK activation [15]. In hippocampal primary cultures ERK1/2 phosphorylation was induced 5 minutes after Neurturin (50 ng/mL) application (Figure 1(a)). The effect was sensitive to MEK blocker U0126 (20 *μ*M). Similarly to our previous results with BDNF [3], Neurturin induced Egr4 expression: Egr4 mRNA levels were 1.6 ± 0.2-fold higher than in control one hour after Neurturin application (Figure 1(b)).

Although acting through a very different type of tyrosine kinase receptor, Neurturin, similarly to BDNF, significantly upregulated KCC2 protein expression at 10 and 50 ng/mL (129 ± 4% and 171 ± 16%; 10 and 50 ng/ml resp.  Figure 1(c)). Interestingly, the effect of Neurturin also had a tendency to decline with culture maturation. The strongest up-regulation of KCC2 was observed in two-weeks-old cultures (130 ± 7% of control; Figure 1(d)). 

The developmental change in the effect of the neurotrophic factors could be caused by a difference in the expression level of corresponding receptors. Thus we monitored the expression of Neurturin coreceptors RET and GFR*α*2 in dissociated cultures at div4, div11, and div18 by semiquantitative RT-PCR (Figure 1(e)). The PCR results showed that mRNAs for these receptors were detectable at all ages investigated. When compared to div4, GFR*α*2 mRNA was down-regulated to 32 ± 16% at div25. This change in GFR*α*2 expression may have a role in the developmental differences in the effect of Neurturin on KCC2 expression.

These data provide evidence that Neurturin, similarly to BDNF, is able to enhance endogenous KCC2 expression *in vitro* during early postnatal period in a dose-dependent manner. The developmental change in trophic factor-mediated regulation of KCC2 expression may be a consequence of corresponding changes in trophic factor receptor expression.

### 3.2. Neurturin Activation of KCC2 Proximal Promoter Region is Dependent on MEK Phosphorylation

**Figure 2:**
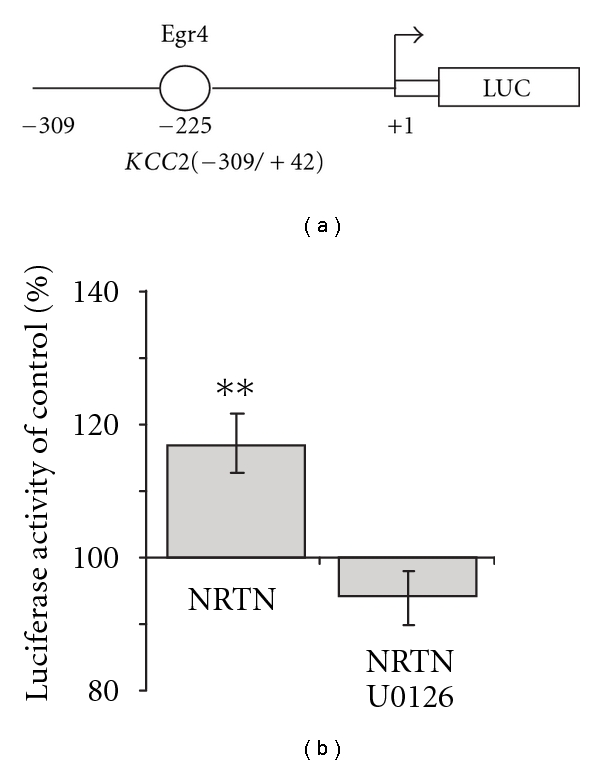
Neurturin-induced activation of KCC2 proximal promoter region. (a) Schematic drawing of the luciferase construct carrying the KCC2 proximal promoter region. The construct contains the luciferase reporter gene under control of a short (309 bp) upstream proximal part of the KCC2 promoter sequence. (b) Normalized luciferase activity 2–4 hours after application of 50 ng/mL Neurturin in div7 hippocampal neuronal cultures transfected with the KCC2 (−309/+42) construct (*n* = 4; **P < 0.01). Error bars represent SEM.

Using the luciferase (Luc) reporter construct *KCC2(−309/+42) *driven by the proximal promoter region of KCC2 (Figure 2(a)) we performed experiments on hippocampal primary cultures aiming to further study Egr4 involvement in the neurotrophic factor-induced KCC2 up-regulation (for detailed scheme of construct generation see [16]).

Dissociated neurons were transfected with *KCC2(−309/+42) *at div4 and treated with 50 ng/mL Neurturin two days after transfection. Two to four hours after the trophic factor application, culture lysates were analyzed for Luc activity (Figure 2(b)). We observed substantial increase in KCC2 promoter activity in cultures treated with Neurturin (117 ± 5% of nontreated controls). The Neurturin-induced increase in KCC2 promoter activity was abolished by MEK1/2 inhibitor U0126 (20 *μ*M).

### 3.3. Expression Pattern of the Neurturin Receptors GFR*α*2 and RET in the Early Postnatal Hippocampus

In contrast to TrkB receptor, in which expression is well characterized in early postnatal brain [17], detailed data on Neurturin co-receptors RET and GFR*α*2 expression in the hippocampus are scarce. Two studies addressed the question of GFR*α2* and RET expression in developing hippocampus by RT PCR and *in situ* hybridization [18, 19]. Both studies showed that after birth GFR*α*2 mRNA expression reaches maximum at around P5 and it is downregulated during later development. The *in situ* hybridization study [18, 19] also demonstrated that at P4 a prominent RET and GFR*α*2 mRNA expression was localized to CA3 pyramidal layer while GFR*α*1 mRNA was only weakly abundant there. 

**Figure 3:**
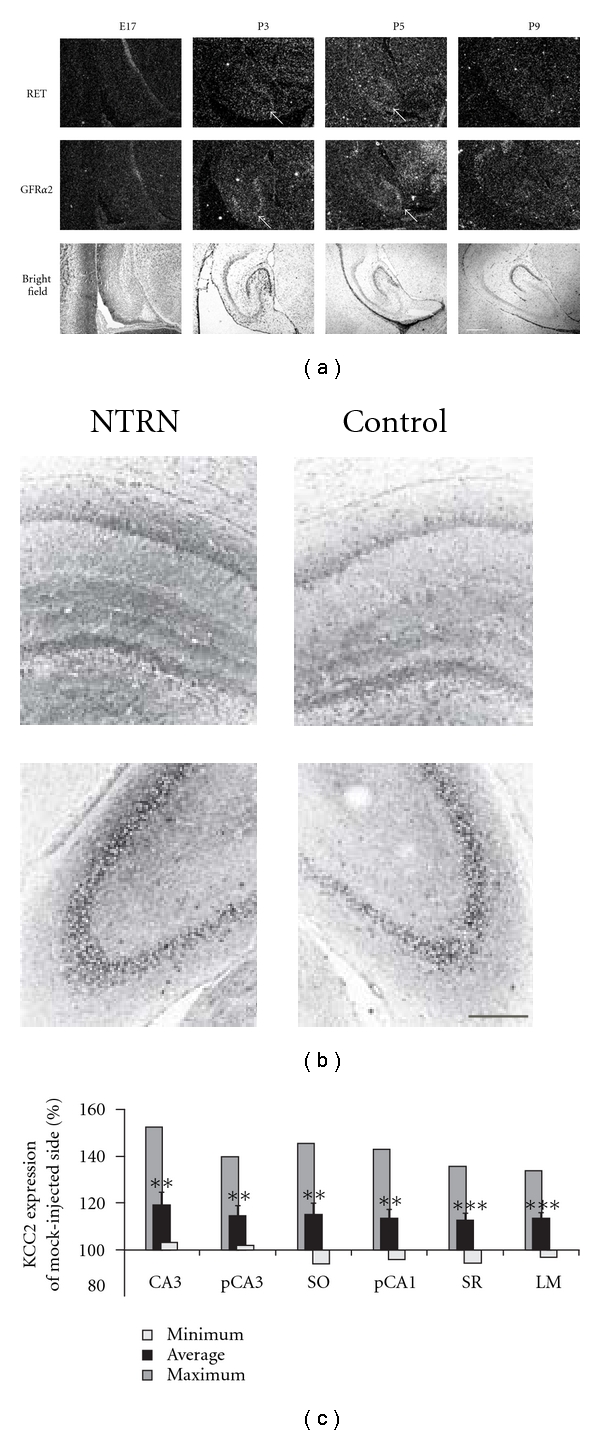
Regulation of KCC2 expression by Neurturin *in vivo*. (a) RET and GFR*α*2 mRNA expression was detected by *in situ* hybridization. Note the accumulation of RET and GFR*α*2 at P3 and P5 in the CA3 region of the hippocampus (marked with arrowheads). Scale bar = 500 *μ*m. (b) Representative immunofluorescent stainings of KCC2 expression in Neurturin-treated and control hippocampi 3 days after injection. Neurturin (1 *μ*g) was used for injection in right-side hippocampus of P5 rat while contralateral hippocampus was injected with saline solution. (c) Summary results of KCC2 immunostaining intensity for different layers of CA1 and CA3 in Neurturin-injected hemisphere (*n *= 16–21). KCC2 intensity was measured in 6 sections at various distances from injection site. The effect of Neurturin injection was uneven along the dorsal-ventral axis. Quantification was made for the following layers: CA3 is whole area of CA3; pCA3 and pCA1 are pyramidal layers of CA3 and CA1, respectively; SO is stratum oriens; SR is stratum radiatum and LM is stratum lacunosum-moleculare. In each section and each layer KCC2 intensity in Neurturin-injected hemisphere was normalized to the corresponding value in control hemisphere. Then for each layer the maximum, the minimum, and the average values of KCC2 intensity along the dorsal-ventral axis were calculated (**P < 0.01, ***P < 0.001). Error bars represent SEM. Scale bar = 300 *μ*m.

We analyzed the temporal and spatial pattern of GFR*α*2 and RET mRNA expression during early postnatal development in our conditions in order to estimate the best optimal time point to test the effect of Neurturin *in vivo*. *In situ* hybridization of consecutive sagital rat brain sections at E17, P3, P5, and P9 (Figure 3(a)) showed that RET and GFR*α*2 signals were relatively low but most prominent at P3 and P5. RET mRNA expression showed a general dispersed pattern. Interestingly, GFR*α*2 mRNA was most prominent in the pyramidal layer of the CA3 region (white arrowheads).

### 3.4. Neurturin Increases KCC2 Expression in P5-P8 Mice Hippocampus In Vivo

We investigated the effect of intrahippocampal Neurturin injections on KCC2 expression. In accordance with receptor expression data, Neurturin was injected in hippocampi of P5 rats. In the initial series of experiments two doses of Neurturin were tested: 100 ng and 1 *μ*g. The lower dose of Neurturin had no significant effect on KCC2 expression (data not shown). All further experiments were performed using 1 *μ*g of Neurturin. 

Single injection of Neurturin produced a significant increase in KCC2 expression (Figures 3(b) and 3(c)). KCC2 levels were analyzed three days after the Neurturin injection by immunostaining. Representative KCC2 immunostaining of the areas with maximal Neurturin effect are presented in Figure 3(b). Summarized data for three independent experiments are shown in Figure 3(c). KCC2 up-regulation after Neurturin injection was significant in all hippocampal areas analyzed. The maximal effect of Neurturin (150% of control) was observed in CA3 area.

Taken together, the *in vivo* experiments indicate that within three days after a single application of Neurturin in developing hippocampus, there is a substantial increase in the level of KCC2 expression.

## 4. Discussion

Identification of the intracellular cascades involved in KCC2 regulation is crucial for understanding the mechanisms rendering long-term plastic changes in GABA_A_ mediated transmission during development and trauma. Both BDNF and Neurturin can activate similar intracellular cascades that include Shc/Frs2 which in turn triggers the MAPK and AKT signaling [5, 15]. The MAPK pathway is known to be involved in differentiation and maturation whereas AKT is implicated in cell growth and survival. The results obtained in the present study clearly show that it is activation of the MAPK intracellular pathway that is crucial for both BDNF—[3] and Neurturin—induced expression of KCC2 in immature neurons. Suppression of MAPK signaling by the specific MEK inhibitor U0126 resulted in a significant block of BDNF-[3, 20] and Neurturin—induced KCC2 promoter activation as well as Neurturin-induced Egr4 expression. Our previously published results showed that in adult neurons activation of mutant TrkB receptor, in which only Shc/Frs2 docking site was preserved, resulted in KCC2 up-regulation [2] thus emphasizing the importance of this pathway not only during development but also under pathophysiological conditions. 

Our previous results demonstrated that early growth response factor Egr4 induced KCC2 transcription [16] and mediated the BDNF-induced KCC2 up-regulation [3] in immature neurons. In the present work we show that similar to the effect of BDNF, Neurturin significantly up-regulates Egr4. This obviously leads to the question whether Egr4 is involved in the Neurturin-induced KCC2 up-regulation. The crucial role of Egr4 in this process is particularly indicated by the experiment where Neurturin displays a significant activation of the proximal promoter region of KCC2 that carries the binding site for Egr4. Consistent with the *in vitro* results, *in vivo* intrahippocampal injection of Neurturin produced a significant up-regulation of KCC2 protein in neonatal rat hippocampus. 

The family of GDNF ligands are potent survival promoting trophic factors that act primarily through the interaction with GFR*α* coreceptors and signal through the activation of the receptor tyrosine kinase RET. Resent results suggest that, additionally, this family of trophic factors could signal through alternative signaling pathways [21]. The temporal and spatial effect of Neurturin correlated both *in vitro* and *in vivo* with the expression profile of RET and GFR*α*2, suggesting that this effect was mediated trough the specific activation of these receptors. More detailed analysis of the effect of Neurturin on KCC2 expression in GFR*α*2−/− mice in the future will give a more decisive answer to whether this effect is mediated trough GFR*α*2/RET.

In conclusion the present results are in agreement with a central role of the intracellular MAPK/ERK pathway as convergent point for different parallel extracellular cues. Activation of the MAPK pathway by these extracellular signals induces immediate early gene Egr4 expression that in turn stimulates KCC2 up-regulation. These may lead to increased Cl^−^  extrusion efficiency causing the maturation of GABA_A_ mediated responses. These results suggest that there may be several extracellular signals able to induce KCC2 up-regulation in developing neurons. One question for the future is whether Neurturin and BDNF have synergistic action *in vivo* to regulate KCC2 during development. Another important question raised by the present study is whether parallel mechanisms regulating KCC2 expression are in place also under pathophysiological conditions.

## Figures and Tables

**Table 1:** 

Product	Forward	Reverse
RET	5′-AGGACCACACATCACTTTGAG-3′	5′-ATGAAAGGGTACTGACCATGG-3′
GFR*α*2	5′-TATTGGAGCATCCATCTGGG-3′	5′-AGCAGTTGGGCTTCTCCTTG-3′
GAPDH	5′-GCAAAGTGGAGATTGTTGCCAT-3′	5′-CCTTGACTGTGCCGTTGAATTT-3′

**Table 2:** 

Product	Forward	Reverse
Egr4	5′-TCTCTCCAAGCCCACCGAAG-3′	5′-AACCGCCTGGATGAAGAAGC-3′
GAPDH	5′-GCAAAGTGGAGATTGTTGCCAT-3′	5′-CCTTGACTGTGCCGTTGAATTT-3′

## Abbreviations

Shc: Src homology 2 domain containing transforming protein
FRS2: FGF receptor substrate 2
PLC*γ*: Phospholipase C*γ*
MAPK: Ras/mitogen activated protein kinase
PI3K: Phosphatidylinositol 3- kinase
AKT: Cellular homolog of the viral oncogene v-Akt
TrkB: Tyrosine receptor kinase B
GAPDH: Glyceraldehyde-3-phosphate dehydrogenase
BDNF: Brain derived neurotrophic factor
NRTN: Neurturin
Egr4: Early growth response 4
div: Days in vitro
GDNF: Glial cell line-derived neurotrophic factor
RET: Rearranged during transfection
GRFalpha2: GDNF family receptor alpha 2
MEK: MAPK/ERK kinase
SEM: Standard error of the mean
SOS: Son of sevenless
Grb: Growth factor receptor-bound protein
MAPK: Mitogen-activated protein kinase
Erk: Extracellular signal-regulated kinase
PIP2: Phosphatidylinositol 4,5-biphosphate
DAG: Diacyl glycerol
PKC: Protein kinase C.
